# A two-dose viral-vectored *Plasmodium vivax* multistage vaccine confers durable protection and transmission-blockade in a pre-clinical study

**DOI:** 10.3389/fimmu.2024.1372584

**Published:** 2024-04-30

**Authors:** Yutaro Yamamoto, Camila Fabbri, Daiki Okuhara, Rina Takagi, Yuna Kawabata, Takuto Katayama, Mitsuhiro Iyori, Ammar A. Hasyim, Akihiko Sakamoto, Hiroaki Mizukami, Hisatoshi Shida, Stefanie Lopes, Shigeto Yoshida

**Affiliations:** ^1^Laboratory of Vaccinology and Applied Immunology, Kanazawa University School of Pharmacy, Kanazawa University, Kanazawa, Ishikawa, Japan; ^2^Instituto Leônidas & Maria Deane/Fiocruz Amazônia, Laboratório de Diagnóstico e Controle e Doenças Infecciosas da Amazônia, Manaus, Amazonas, Brazil; ^3^Fundação de Medicina Tropical Dr. Heitor Vieira Dourado, Unidade de Pesquisa Clínica Carlos Borborema - UPCCB, Manaus, Amazonas, Brazil; ^4^Department of Pharmaceutical Sciences, Musashino University, Tokyo, Japan; ^5^Division of Genetic Therapeutics, Jichi Medical University, Shimono, Tochigi, Japan; ^6^Laboratory of Primate Model, Research Center for Infectious Diseases, Institute for Frontier Life and Medical Science, Kyoto University, Kyoto, Japan

**Keywords:** malaria, vaccine, *Plasmodium vivax*, PvCSP, Pvs25, LC16m8Δ, adeno-associated virus

## Abstract

Among *Plasmodium* spp. responsible for human malaria, *Plasmodium vivax* ranks as the second most prevalent and has the widest geographical range; however, vaccine development has lagged behind that of *Plasmodium falciparum*, the deadliest *Plasmodium* species. Recently, we developed a multistage vaccine for *P. falciparum* based on a heterologous prime-boost immunization regimen utilizing the attenuated vaccinia virus strain LC16m8Δ (m8Δ)-prime and adeno-associated virus type 1 (AAV1)-boost, and demonstrated 100% protection and more than 95% transmission-blocking (TB) activity in the mouse model. In this study, we report the feasibility and versatility of this vaccine platform as a *P. vivax* multistage vaccine, which can provide 100% sterile protection against sporozoite challenge and >95% TB efficacy in the mouse model. Our vaccine comprises m8Δ and AAV1 viral vectors, both harboring the gene encoding two *P. vivax* circumsporozoite (PvCSP) protein alleles (VK210; PvCSP-Sal and VK247; -PNG) and P25 (Pvs25) expressed as a Pvs25–PvCSP fusion protein. For protective efficacy, the heterologous m8Δ-prime/AAV1-boost immunization regimen showed 100% (short-term; Day 28) and 60% (long-term; Day 242) protection against PvCSP VK210 transgenic *Plasmodium berghei* sporozoites. For TB efficacy, mouse sera immunized with the vaccine formulation showed >75% TB activity and >95% transmission reduction activity by a direct membrane feeding assay using *P. vivax* isolates in blood from an infected patient from the Brazilian Amazon region. These findings provide proof-of-concept that the m8Δ/AAV1 vaccine platform is sufficiently versatile for *P. vivax* vaccine development. Future studies are needed to evaluate the safety, immunogenicity, vaccine efficacy, and synergistic effects on protection and transmission blockade in a non-human primate model for Phase I trials.

## Introduction

Malaria, the most important parasitic disease of humans, is caused by parasites of the *Plasmodium* genus and is transmitted by female *Anopheles* (*An.*) mosquitoes (1). Most human malaria cases stem from infection by either of two *Plasmodium* parasite species: *P. falciparum* and *P. vivax*. Research efforts have primarily centered on *P. falciparum* malaria because of its high mortality rates in sub-Saharan Africa. However, *P. vivax* malaria affects more of the population across a wider geographical range (95 countries), with 2.85 billion people at risk of disease every year (2). Furthermore, recent studies documented severe cases of *P. vivax* in children, including cerebral malaria, severe anemia, acute respiratory syndrome, and thrombocytopenia (3–5).

In addition to its broad geographic distribution, including some of the most densely populated regions on Earth, another factor that makes *P. vivax* particularly difficult to control is its ability to remain within hepatocytes in a latent/inactive liver form, called hypnozoites, which can become active weeks, months, or even years after the initial mosquito-transmitted infection. Upon activation, the parasites multiply, and the generated merozoites leave hepatocytes, leading to a relapse of the symptomatic blood-stage infection (6). The relapse causes symptoms without any further sporozoite infection via mosquito transmission. Therefore, relapses contribute to the burden of the disease because, after hypnozoite reactivation, the blood parasite can infect mosquitoes and be transmitted to new susceptible hosts. Despite the significance of effective interventions being acknowledged, the absence of long-term *in vitro* culture systems using red blood cells, appropriate animal models, insufficient research funds, and the complex lifecycle of the parasite have impeded progress in the development of a potent vaccine (7, 8).

For *P. vivax* vaccine development, pre-erythrocytic vaccines (PEV) targeting sporozoite surface proteins might block hepatocyte invasion and the establishment of hypnozoites. This strategy may have an anti-infection effect and decrease the risk of recurrent infection (9). The primary candidate for PEV is the circumsporozoite protein (CSP), present on the surface of all types of *Plasmodium* sporozoites and is currently, the most advanced contender for an effective malaria vaccine (10). A challenge hindering the progress of a *P. vivax* CSP (PvCSP)-based vaccine is the higher diversity observed within the PvCSP molecule compared with *P. falciparum*. Specifically, three distinct PvCSP alleles (VK210, VK247, and *P. vivax*-like) have been identified globally, each differing in the central repetitive region of the molecule. *P. vivax* infections are widespread worldwide, with VK210 and VK247 predominating. Several studies have focused on the development of a universal vaccine that targets the three different PvCSP alleles. However, a highly effective vaccine against infectious diseases has not been developed to date (11, 12). Considering the global distribution of the predominant VK210 and VK247 variants, it would be ideal to develop a PvCSP-based vaccine capable of inducing protective immune responses against both allelic forms at least (13).

The *P. falciparum* R21 vaccine based on *P. falciparum* CSP (PfCSP), developed by the University of Oxford, is considered a next−generation RTS,S−like vaccine. Notably, in a Phase II trial conducted in Burkina Faso, the vaccine demonstrated efficacy of up to 77% against clinical malaria over 1 year (14). Research led by Arturo Reyes–Sandoval applied R21 technology based on a virus-like particle to generate a *P. vivax* vaccine candidate, Rv21 (15). This vaccine contained the VK210 and VK247 sequences of PvCSP conjoined with hepatitis B virus-like particles, and was produced using the yeast *Pichia pastoris*. When administered in conjunction with Matrix-M adjuvant, the Rv21 vaccine demonstrated durable and absolute protection against sporozoites in mice. Additionally, the antibodies produced in response to both variants effectively identified PvCSP on the surface of *P. vivax* sporozoites (15). In another approach, Herrera et al. reported that PvCSP-derived long synthetic peptides with Montanide ISA720 or Montanide ISA51 were safe, well-tolerated, and induced sterile protection in 36.6% of naive volunteers and 27.3% of semi-immune volunteers against Controlled Human Malaria Infection (CHMI) in Phase I clinical trials in Colombia (16).

Malaria parasites pass through multiple phases throughout their intricate life cycle. To diminish the individual burden of disease and spread of malaria in the general population, a vaccine that targets the multiple stages of malaria’s lifecycle might be a more effective approach than one that focuses on a single stage. Such a multistage vaccine strategy would be particularly effective against *P. vivax*, which includes a dormant hypnozoite stage in its life cycle. We recently developed a vaccine platform using viral vectors, including a highly attenuated strain of vaccinia virus, LC16m8Δ (m8Δ)–a genetically stable variant of the efficacious Japanese smallpox vaccine LC16m8, and adeno-associated virus (AAV), commonly utilized in gene therapy for humans. The multistage vaccine for *P. falciparum*, incorporating genes for the circumsporozoite protein PfCSP and ookinete protein Pfs25, fused into a single protein, induced sterile protection and halted transmission, maintaining prolonged robust immune responses and protective efficacy (17).

In the present study, we generated a *P. vivax* multistage m8Δ/AAV vaccine harboring the genes encoding PvCSP and Pvs25 as a fusion protein and showed the feasibility and versatility of the m8Δ/AAV vaccine platform for *P. vivax* vaccine development. The heterologous m8Δ-prime/AAV-boost vaccination regimen in murine models achieved complete protection (100%) against PvCSP-transgenic *P. berghei* sporozoites and demonstrated up to 95% efficacy in preventing malaria transmission. This was confirmed using a direct membrane feeding assay (DMFA) using parasites from naturally *P. vivax*-infected individuals in malaria-endemic regions. Our vaccine platform provides adaptability and superior qualities for managing malaria caused by *P. falciparum* and *P. vivax*. We propose that the m8Δ/AAV *P. vivax* multistage vaccine will have a significant role in fulfilling the ambitious objectives set forth in the malaria vaccine technology roadmap, aiming for sustained sterile immunity and a high degree of transmission-blocking (TB) effectiveness.

## Materials and methods

### Cells and viruses

HEK293T cells and baby hamster kidney (BHK) cells were cultured in Dulbecco’s modified Eagle’s medium (DMEM) (Gibco, Life Technologies) supplemented with 10% heat-inactivated fetal bovine serum (FBS), non-essential amino acids, penicillin, and streptomycin. Rabbit kidney RK13 cells were cultured in RPMI 1640 medium (Gibco, Life Technologies) supplemented with 10% heat inactivated FBS, penicillin, and streptomycin. Canarypox virus and m8Δ were previously described (18).

### Parasites and animals

The genetically modified *P. berghei* parasite lines utilized in this study, engineered PvCSP-VK210 and -VK247, were initially developed by the Laboratory of Vaccinology and Applied Immunology, Kanazawa University (19, 20). *An. stephensi* mosquitoes (SDA 500 strain) were used as hosts to these transgenic parasites after feeding on parasite-infected 6-week-old BALB/c mice. Female BALB/c mice, acquired from Japan SLC (Hamamatsu, Shizuoka, Japan), were consistently used in the present study when they reached the age of 6–8 weeks.

### *Anopheles* spp. mosquito colonies

*An. stephensi* females were reared at the Laboratory of Vaccinology and Applied Immunology of Kanazawa University in Japan. The colony was kept at a temperature of 20°C and a relative humidity of 60%. Larvae were hatched in room temperature water and fed with fish food (KAMIHATA®). The larvae were allowed to pupate and emerge into adults in an enclosed mesh-covered cage with water and 5% fructose. Female mosquitoes were used for experiments at 7–10 days old.

*An. aquasalis* and *An. darlingi* females were reared at the Laboratory of Medical Entomology at Fundação de Medicina Tropical Dr. Heitor Vieira Dourado (FMT-HVD) in Manaus, Brazil. The colonies were kept at a temperature of 24°C–26°C and a relative humidity of 70%–80%. Larvae were hatched in room temperature water and fed with fish food (TetraMin®). The larvae were allowed to pupate and emerge into adults in an enclosed mesh-covered cage with water and 10% glucose for *An. aquasalis* or 15% of honey for *An. darlingi*. Female mosquitoes used for experiments were 3–6 days old (21, 22). The glucose or honey solution was removed 12–24 h prior to the DMFA to starve the mosquitoes.

### Viral vector construction

The recombinant vaccinia virus m8Δ-Pv(P7.5-s25-CSP-VK210/247)-HA vector, was generated by homologous recombination for foreign gene insertion into the *HA* gene locus as described previously (23). This m8Δ virus is designed to express proteins under the control of the p7.5 promoter. BHK cells were infected with the canarypox virus at a multiplicity of infection (MOI) of 10 and then transfected with the transfer plasmid vector, pVR1-Pv(P7.5-s25-CSP-VK210/247)-HA, and the m8Δ genomic DNA using lipofectamine LTX (Thermo Fisher Scientific, Waltham, MA, USA). The transfected cells were cultured in DMEM supplemented with 10% FBS for one day. The culture medium and cells were then collected and freeze-thawed five times to prepare recombinant m8Δ-Pv(P7.5-s25-CSP-VK210/247)-HA. RK13 cell monolayers were inoculated with each recombinant m8Δ-Pv(P7.5-s25-CSP-VK210/247)-HA, followed by plaque purification. Loss of HA expression was confirmed using peripheral blood from a white leghorn (Shimizu Laboratory Supplies, Kyoto, Japan), as previously described (24). Insertion of the Pv(P7.5-s25-CSP-VK210/247) gene into the m8Δ-HA region was confirmed by PCR using the following primer pair:

5′-CACCCATATGTACCATAGCATCGAGTTGATATCTATCGG-3′ (F1) and
5′-GCAGGTGTCCTCCTTCAGTGTATAGCC-3′ (R1).

For the generation of AAV1-Pv(s25-CSP-VK210/247), the gene cassette encoding Pfs25-PfCSP was excised from the pENTR-Pvs25-PvCSP(VK210/247)-G2-WPRE plasmid by digestion with *Mun* I/*Xho*I and then inserted into the *EcoR*I and *Xho*I sites of the pAAV-sPfs25-sPfCSP-G2 vector to construct pAAV-Pvs25-CSP(VK210/247), which facilitated the production of AAV1-Pv(s25-CSP-VK210/247). This was achieved by transfecting HEK293T cells with the plasmid, as described in a previous study (25, 26).

### Immunoblotting

HEK293T cells were infected with m8Δ-Pv(P7.5-s25-CSP-VK210/247)-HA at an MOI of 5 or with AAV1-Pv(P7.5-s25-CSP-VK210/247) at an MOI of 10^5^. Cell lysates were collected using Laemmli buffer 24 or 48 h post-infection and subjected to immunoblotting as described previously (27). The cell lysates were electrophoresed on 10% sodium dodecyl sulfate polyacrylamide gel (SDS-PAGE) under reducing conditions for probing with the anti-PvCSP-VK210 monoclonal antibody (mAb) 2F2 or anti-PvCSP-VK247 mAb 2E10E9. Under non-reducing conditions, anti-Pvs25 mAb N1-1H10 was used. Each blot was incubated with goat anti-mouse IRDye 800 conjugated secondary antibodies (Rockland Immunochemicals, Limerick, PA, USA), and then visualized using an Odyssey infrared imager (LI-COR, Lincoln, NE, USA) (28).

### Immunofluorescence assay

IFAs were performed to evaluate protein expression using fluorescently labeled antibodies. Rabbit kidney (RK) cells at a concentration of 5 × 10^4^ cells per well, were infected with the m8Δ vaccine through a serial dilution process. When virus plaques were formed 72 h post-infection, live cell staining was performed. In brief, R-Phycoerythrin LK23-conjugated anti-PvCSP-VK210 mAb 2F2, Fluorescein LK01-conjugated anti-PvCSP-VK247 mAb 2E10E9 or HiLyte Flour™ 647 LK13-conjugated anti-Pvs25 mAb N1-1H10 was directly added to the plaque forming cells, and a BZ-X710 fluorescence microscope (Keyence Corp., Tokyo, Japan) was used for image acquisition.

### Immunization

For prime immunization, 1 × 10^7^ plaque-forming units (PFU) of m8Δ-Pv(P7.5-s25-CSP-VK210/247) were administered by tail scarification (29) as priming, and 1 × 10^10^ viral genomes of AAV1-Pv(s25-CSP-VK210/247) were intramuscularly administered 6 weeks after priming. Negative control animals were injected with PBS or m8Δ-prime and AAV1-boost after a 6-week interval (17).

### Enzyme-linked immunosorbent assay

**Figure 1:**
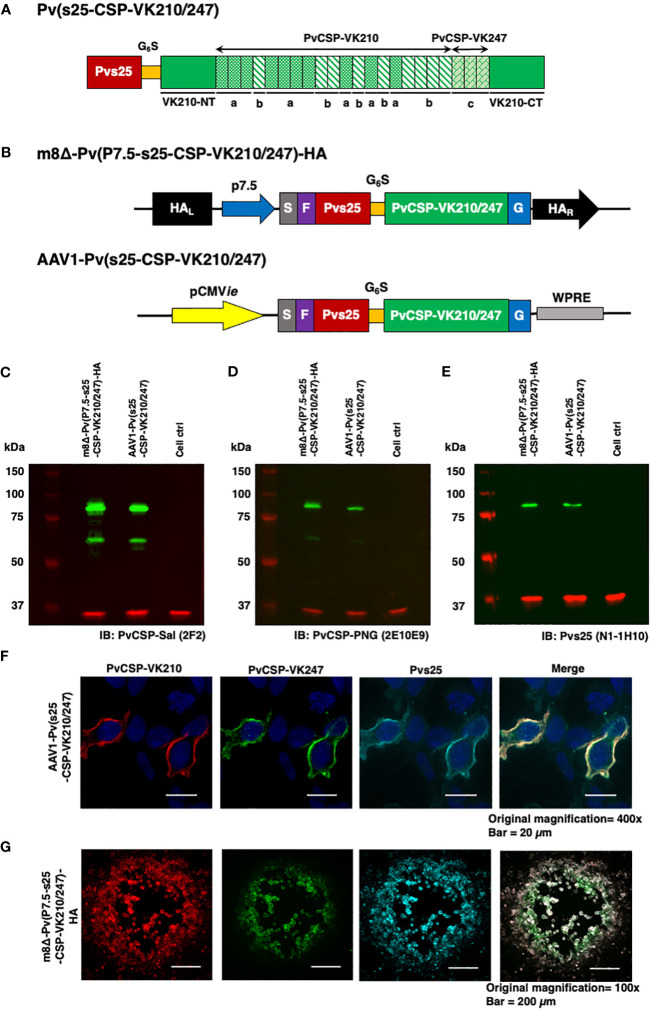
Construction of the m8Δ/AAV1 vaccine. **(A)** The gene cassette encoding the Pvs25-PvCSP fusion protein is shown; the chimeric *pvcsp* gene encoding amino acids 19–373 of PvCSP VK210 (i.e., lacking the N-terminal signal peptide and C-terminal glycosylphosphatidylinositol anchor sequences) and the three repeat sequence units (a, GDRADGQPA; b, GDRAAGQPA; c, GAGNQPGAN) of PvCSP VK247 are fused to the C-terminus of Pvs25. **(B)** The gene cassette was introduced into the AAV1 and m8Δ genomes to generate AAV1-Pv(s25-CSP-VK210/247) and m8Δ-Pv(P7.5-s25-CSP-VK210/247)-HA, respectively. Expression of the *pvcsp-pvs25* fusion gene cassette was driven by the CMV promoter in AAV1 and by the P7.5 promoter in m8Δ. HA, *hemagglutinin* gene; S, gp64 signal sequence; P7.5, 7.5 promoter; pCMV, CMV immediate early promoter; F, FLAG epitope tag; G6S, GGGGGS hinge sequence; G, VSV-G TM; WPRE, woodchuck hepatitis virus posttranscriptional regulatory element. **(C–E)** Analysis of Pvs25-PvCSP fusion protein expression in HEK293T cells transduced with AAV1-Pv(s25-CSP-VK210/247) (MOI = 10^5^) or m8Δ-Pv(P7.5-s25-CSP-VK210/247)-HA (MOI = 5). Cells were lysed and loaded onto a 10% SDS-PAGE gel and immunoblotted with anti-PvCSP VK210 mAb 2F2 **(C)**, anti-PvCSP VK247 mAb 2E10E9 **(D)**, or anti-Pvs25 mAb N1-1H10 **(E)**. **(F, G)** Localization of the Pvs25-PvCSP fusion protein in mammalian cells after transduction with AAV1-Pv(s25-CSP-VK210/247) in HEK293T cells **(F)** or with m8Δ-Pv(P7.5-s25-CSP-VK210/247)-HA in RK13 cells **(G)**. **(F)** After 24 h, the cells were fixed with paraformaldehyde and blocked with 10% normal goat serum. Then, the cells were incubated with R-Phycoerythrin LK23-conjugated anti-PvCSP VK210 mAb 2F2 (red), Fluorescein LK01-conjugated anti-PvCSP VK247 mAb 2E10E9 (green), and HiLyte Flour™ 647 LK13-conjugated anti-Pvs25 mAb N1-1H10 (light blue). Cell nuclei were visualized with DAPI (blue). Original magnification, 100×. Scale bars = 200 µm. **(G)** After 24 h, the infected cells formed plaques and were directly immunostained with R-Phycoerythrin LK23-conjugated anti-PvCSP VK210 mAb 2F2 (red), Fluorescein LK01-conjugated anti-PvCSP VK247 mAb 2E10E9 (green), and HiLyte Flour™ 647 LK13-conjugated anti-Pvs25 mAb N1-1H10 (light blue). Original magnification, 400×. Scale bar = 20 μm.

Serum samples were collected from the tail veins of mice. For the analysis of humoral immunity, blood was collected continuously from the tail vein for 8 months, and antibody titers were measured by ELISA (**Figure 1**). PvCSP-VK210 and Pvs25 specific IgGs titers were quantified by ELISA as described previously (30). Briefly, the wells of microtiter plates (96-well EIA/RIA plate, Coster®, Kennebunk, CA, USA) were coated with 100 µl of PvCSP-VK210 protein (200 µg/ml), PvCSP-VK247 peptide (200 µg/ml, NH_2_-GAGNQPGANGAGNQPGANGAGNQPGAN-COOH, Eurofins, Kyoto, Japan), and Pvs25 protein (400 µg/ml) overnight at 4°C and then blocked with PBS containing 1% BSA. All proteins used for this study were produced by the *E. coli* system (19). Tail blood samples were serially diluted and then added to the wells, where they were incubated at room temperature for 1 hour. Subsequent to washing, horseradish peroxidase (HRP)-conjugated goat anti-mouse IgG (H+L) antibodies (Bio-Rad, Tokyo, Japan) were included in the wells. A substrate solution for peroxidase containing H_2_O_2_ and 2,2′-and-bis(3-ethylbenzothiazoline-6-sulfonate) was used to develop the plates. The endpoint titers were denoted as the inverse of the most diluted sample that yielded an optical density of 0.15 units at 414 nm, surpassing the optical density of the negative controls, which was below 0.1 unit. Prior to vaccination, all mice in the study were confirmed to be seronegative.

### Challenge infection with PvCSP transgenic sporozoites

Mice were i.v. challenged with PvCSP-VK210/Pb (19) or PvCSP-VK247/Pb (31) sporozoites resuspended in RPMI 1640 medium. Sporozoites were prepared as described previously (31). Each mouse was administered 100 µl of media containing 1000 sporozoites via the tail vein. The progression of malaria infection was monitored on days 4–14 by Giemsa staining of thin blood smears obtained from the tail. Protection was characterized by the complete absence of blood-stage parasitemia on day 14 post-challenge. The duration to achieve 1% parasitemia was determined as described previously (27). In addition, mice that had sterile protection against PvCSP-VK210/Pb sporozoites were rechallenged with 1000 PvCSP-VK210/Pb sporozoites. Protective efficacy was defined as the complete absence of detectable blood-stage parasites in peripheral blood smears until day 14, and a model predicting the time to reach 1% parasitemia was generated.

### *P. vivax*-infected blood sample collection

Samples of *P. vivax*-infected patient blood were obtained via venipuncture and placed in heparinized tubes. Patients were recruited at FMT-HVD, Manaus, Amazonas, based on the following inclusion criteria: adults (age 18 years or older), confirmed diagnosis of *P. vivax* monoinfection by positive microscopy (determined by the presence and percentage of infected red blood cells within at least 10,000 observed red blood cells in a Giemsa-stained peripheral blood smear), and positive result from rapid diagnostic tests (Tokyo Future Style, Tokyo, Japan). Percentages of parasitemia and gametocytemia are shown in **Supplementary Table S1**. Patients who had received any antimalarial treatment in the last 30 days were excluded. Following *P. vivax*-infected blood collection, all patients received treatment in accordance with the Brazilian Malaria Treatment Guidelines (32).

### Direct membrane feeding assay of blood from *P. vivax*-infected patients from Brazil

Field-relevant TB efficacy was determined by DMFAs of blood from naturally infected patients (18–60 years old) from the Brazilian Amazon, as described previously (33). Patients visiting malaria clinics in FMT-HVD were examined microscopically for malaria infection using thick blood smears stained with Giemsa stain and rapid diagnostic tests (Tokyo Future Style, Tokyo, Japan). Upon diagnosis with *P. vivax* malaria, patients were invited to contribute blood samples for the DMFA. After blood collection, all malaria patients were treated at the FMT-HVD according to treatment guidelines provided by the Brazilian Health Ministry (32). The DMFA consisted of a membrane feeding system connected to 37°C water circulation with a different input for the blood to feed the mosquitoes. The collected blood was centrifuged, plasma removed, and erythrocytes were washed three times with an equal volume of RPMI 1640. Pelleted erythrocytes were mixed with inactivated human sera, along with the relevant mouse sera (collected post-boost after 28 days, n = 10), or control sera at different concentrations to achieve a final hematocrit of 50%, and were fed to *An. darlingi* or *An. aquasalis* at the desired concentration via a Parafilm® membrane over a 120-minute period. After 28 days, the post-boost serum concentrations examined were 1:5, 1:10, and 1:50 (100 µl, 50 µl, and 10 µl of immune serum, respectively, plus 250 µl of the erythrocyte pellet and complete test samples up to 500 µl with inactivated human AB serum for each group) (**Supplementary Table 2**). Negative control sera were obtained from a group of unimmunized mice, with sera pooled from 30 control mice. Non-blood-fed mosquitoes were removed from the cage after feeding. The engorged mosquitoes were subsequently kept at 26°C and 80% relative humidity. After a 7-day period, the midguts from 8 to 38 mosquitoes per group were dissected, oocysts were tallied, and the number of infected mosquitoes was documented. TRA and TBA were determined using the following formulas: TRA (%) = 100 × [1 − (average number of oocysts in the test group/average number of oocysts in the control group)]; and TBA (%) = 100 × [1 − (proportion of mosquitoes with no oocysts in the test group/proportion of mosquitoes with oocysts in the control group)] (34).

### Statistical analysis

To assess the protective efficacy of the m8Δ/AAV1-Pv(s25-CSP-VK210/247) vaccine, survival analyses at 1% parasitemia of the protected and infected mice in the vaccinated group were compared with those in the PBS or naïve control groups by the log-rank (Mantel-Cox) test to determine statistical significance. To calculate the difference between prime and boost antibody responses, the Mann-Whitney *U-*test was performed to evaluate statistical significance. Oocyst intensity, TRA, and TBA were analyzed by the Kruskal-Wallis test followed by Dunn’s multiple comparisons test using GraphPad Prism software (version 9, San Diego, CA), where p < 0.05 was considered statistically significant. In all figures, *p-*values are shown as follows: *, *p* < 0.05; **, *p* < 0.01; ***, *p* < 0.001; ****, *p* < 0.0001.

## Results

### Construction of m8Δ and AAV1 vaccines and expression of the Pvs25-PvCSP fusion antigen

The *P. vivax* gene construct, *pvs25-pvcsp-VK210/247* (**Figure 1A**), features a connecting hinge peptide composed of six glycines and one serine (Gly_6_Ser_1_) between the *pvs25* and *pvcsp-VK210/247* genes. The expression of the fusion gene was controlled by the cytomegalovirus immediate-early enhancer-promoter (CMV*ie*) in the case of AAV1 and by the 7.5 promoter (P7.5) for m8Δ (**Figure 1B**). Therefore, m8Δ-Pv(P7.5-s25-CSP-VK210/247)-HA and AAV1-Pv(s25-CSP-VK210/247) constructs include the genes for full-length VK210 as well as the three repeat units of the 247 allele type of PvCSP and Pvs25, linked together into a fusion protein. Human embryonic kidney (HEK) 293T cells transduced by m8Δ (lane 1, MOI = 5) or AAV1 (lane 2, MOI = 10^5^,) synthesized the Pvs25-PvCSP-VK210/247 fusion proteins that reacted with anti-VK210 repeat monoclonal antibody (mAb) 2F2, anti-VK247 repeat mAb 2E10E9, and anti-Pvs25 mAb N1-1H10 at a position with a relative *Mr* of 80–100 kDa (**Figures 1C–E**). Furthermore, there were several bands of PvCSP and cleaved products of the fusion protein with a relative *Mr* of 50–55 kDa. The PvCSP-VK210, -VK247, and Pvs25 proteins were also shown to be expressed on the cell surface (**Figures 1F, G**) by an immunofluorescence assay.

### Induction of potent and durable anti-PvCSP and anti-Pvs25 antibody responses

**Figure 2:**
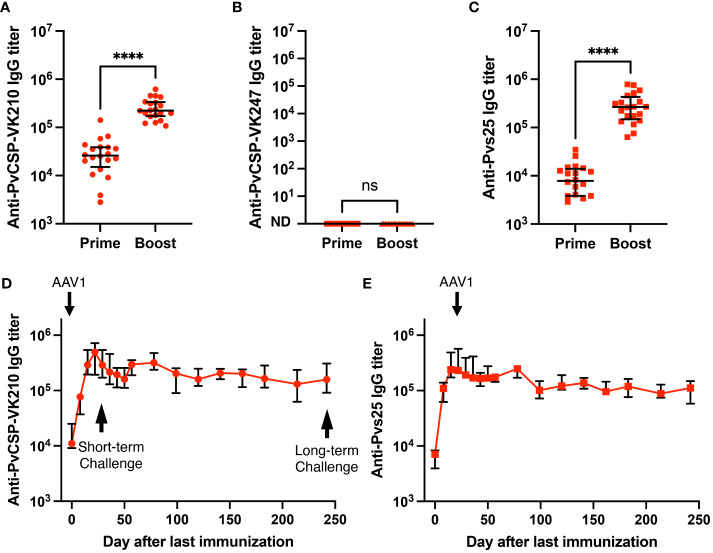
Durability of antibody responses. BALB/c mice (n = 20) were immunized with the m8Δ/AAV1-Pv(s25-CSP-VK210/247) vaccine. **(A–C)** Sera were collected 1 day before the boost immunization (shown as “prime”) and 4 weeks after the boost immunization (shown as “boost”). Antibody titers against PvCSP-VK210 **(A)**, PvCSP-VK247 **(B)**, and Pvs25 **(C)** are shown. Each datapoint represents a single mouse, and horizontal lines indicate the median of antibody titers ± interquartile range. Differences between prime and boost were calculated by the Mann–Whitney U-test. ****p < 0.0001; ns, not significant. **(D, E)** BALB/c mice (n = 5) were immunized and sera were collected weekly up to 248 days. Antibody titers against PvCSP-VK210 **(D)** and Pvs25 **(E)** are shown. The data points indicate the median of antibody titers ± interquartile range.

We have previously shown that the *P. falciparum* m8Δ/AAV1-Pf(s25-CSP) vaccine requires only two immunization doses to induce 100% protection against transgenic *P. berghei* expressing PfCSP sporozoite challenge (23). To test the immunogenicity of the m8Δ/AAV1-Pv(s25-CSP-VK210/247) vaccine, mice were immunized using a heterologous regimen (prime immunization: m8Δ and boost immunization: AAV1) with a 6-week interval, as described previously (23–25). Anti-PvCSP-VK210- and anti-Pvs25-specific IgGs were elicited to titers of 33,279 and 9969 (**Figure 2A**), respectively, 6 weeks after priming, and these titers increased to 266,550 and 309,460, respectively, 4 weeks after boosting (**Figure 2C**). However, no antibodies against the VK247 repeats were detected (**Figure 2B**), indicating that the three VK247 repeat units were less immunogenic than the VK210 repeats (**Figure 1A**). Remarkably, PvCSP-VK210- and Pvs25-specific antibodies were sustained at high-titers for as long as 8 months (**Figures 2D, E**).

### Protective efficacy against transgenic *P. berghei* lines expressing PvCSP

To assess the protective efficacy of the m8Δ/AAV1-Pv(s25-CSP-VK210/247) vaccine, immunized mice in three groups were challenged intravenously with 1000 transgenic sporozoites, as shown in the experimental design (**Supplementary Figure S1**). Briefly, immunized mice were challenged with the PvCSP-VK210/Pb transgenic sporozoites at day 28 (Experiment I; short-term) and day 242 (Experiment II; long-term), or PvCSP-VK247/Pb sporozoites at day 28 (Experiment III).

**Figure 3:**
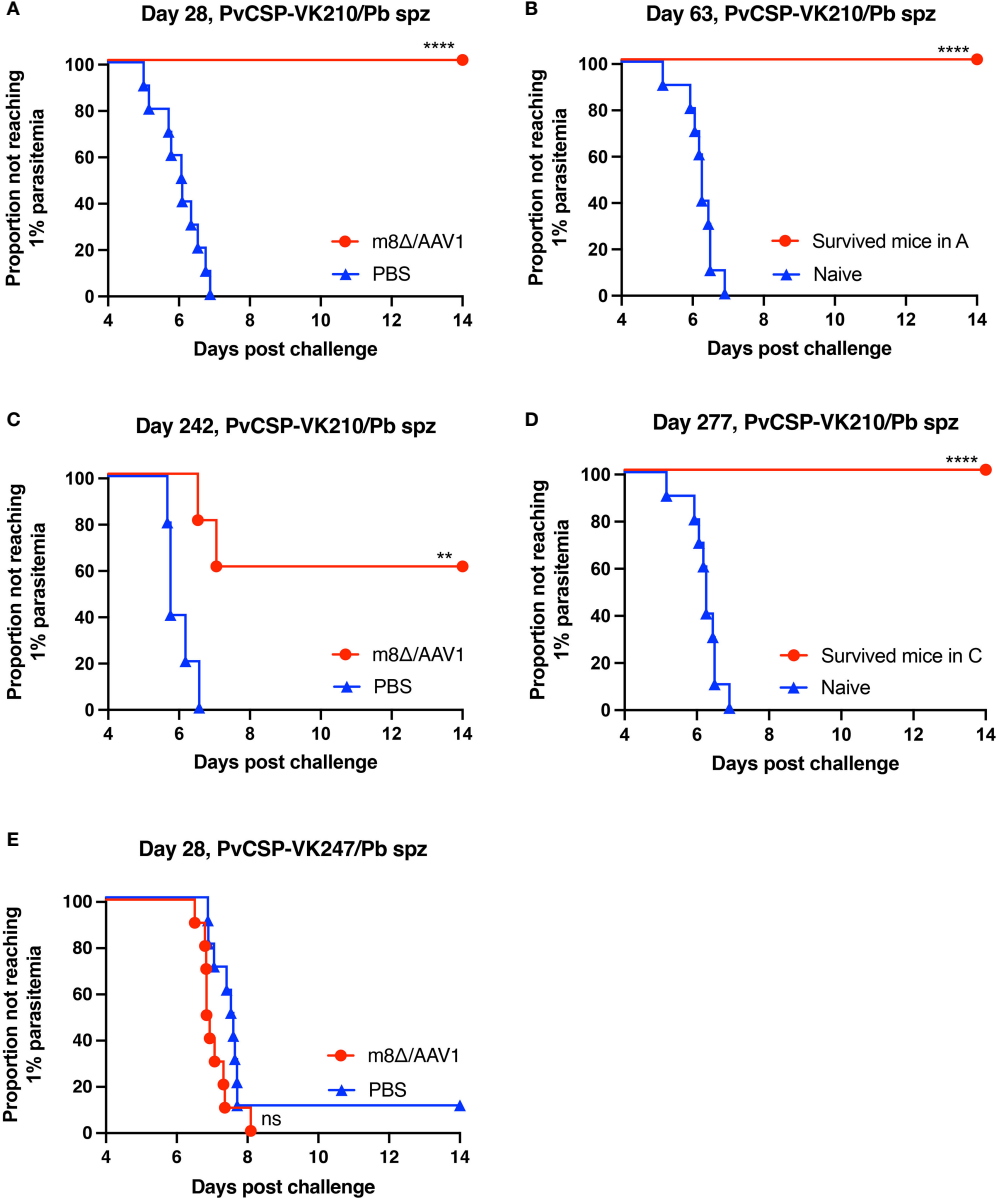
Protective efficacy against sporozoite challenge. BALB/c mice were immunized with the m8Δ/AAV1-Pv(s25-CSP-VK210/247) vaccine. **(A, B)** Experiment I: Mice were challenged with PvCSP-VK210/Pb sporozoites 28 days after immunization with m8Δ/AAV1 (n = 10) or PBS (n = 10) **(A)**. The surviving mice in **(A)** (n = 10) were rechallenged at 63 days (35 days after initial challenge) and compared with naïve mice (n = 10) **(B)**. **(C, D)** Experiment II: Mice were challenged with PvCSP-VK210/Pb sporozoites 242 days after immunization with m8Δ/AAV1 (n = 5) or PBS (n = 5) **(C)**. The surviving mice in **(C)** (n = 3) were rechallenged at 277 days (35 days after initial challenge) and compared with naïve mice (n = 10) **(D)**. Data of naïve control mice in **(B)** and **(D)** were identical in the rechallenge experiments **(B, D)** conducted on the same day. **(E)** Experiment III: Mice were challenged with PvCSP-VK247/Pb sporozoites 28 days after immunization with m8Δ/AAV1 (n = 10) or PBS (n = 10). In all experiments, parasitemia was monitored daily from day 4 after challenge up to day 14. *P-*values were calculated by log-rank (Mantel-Cox) tests versus the control group (PBS or naive). ****p < 0.0001, **p < 0.01.

In Experiment I (short-term), 10/10 mice (100%, *p* < 0.0001) were protected against the PvCSP-VK210/Pb challenge, whereas all control mice were infected (**Figure 3A**). After 35 days, surviving mice were challenged further with PvCSP-VK210/Pb, and 10/10 mice (100%, *p* < 0.0001) were protected against the rechallenge (**Figure 3B**). In Experiment II (long-term), 3/5 mice (60%, *p* < 0.01) were protected against the PvCSP-VK210/Pb challenge (**Figure 3C**). As in Experiment I, all surviving mice (3/3, 100%, *p* < 0.0001) were protected against PvCSP-VK210/Pb rechallenge 35 days after the first challenge (**Figure 3D**). These results indicate that m8Δ/AAV1 vaccine provides potent and long-lasting protective immunity against PvCSP-VK210/Pb. In Experiment III (short-term), 0/10 mice (0%, not significant) were protected against the PvCSP-VK247/Pb challenge (**Figure 3E**). The lack of protective efficacy against PvCSP-VK247/Pb may be related to the lack of induction of anti-PvCSP-VK247 antibodies.

### TB efficacy against field-isolated *P. vivax* in volunteers from the Brazilian Amazon

To evaluate the TB effectiveness of the m8Δ/AAV1-Pv(s25-CSP-VK210/247) vaccine against naturally occurring *P. vivax* at physiological parasite densities, a direct membrane feeding assay (DMFA) was conducted using gametocyte-positive blood samples obtained from four volunteers (Isolate Nos. 1–4) from the Brazilian Amazon. Pooled sera from the immunized groups (collected 4 weeks after the final immunization) were combined with freshly collected gametocyte-positive blood and fed through an artificial membrane to laboratory-reared female *An. aquasalis* for Isolate Nos. 1 and 2 and *An. darlingi* for Isolate Nos. 3 and 4. The parasitemia and gametocytemia of Isolate Nos. 1–4 are summarized in **Supplementary Table S1**. In all four isolates, oocysts were observed in mosquito species in the DMFA, as well as after supplementation with sera from mice that received the PBS control.

**Figure 4:**
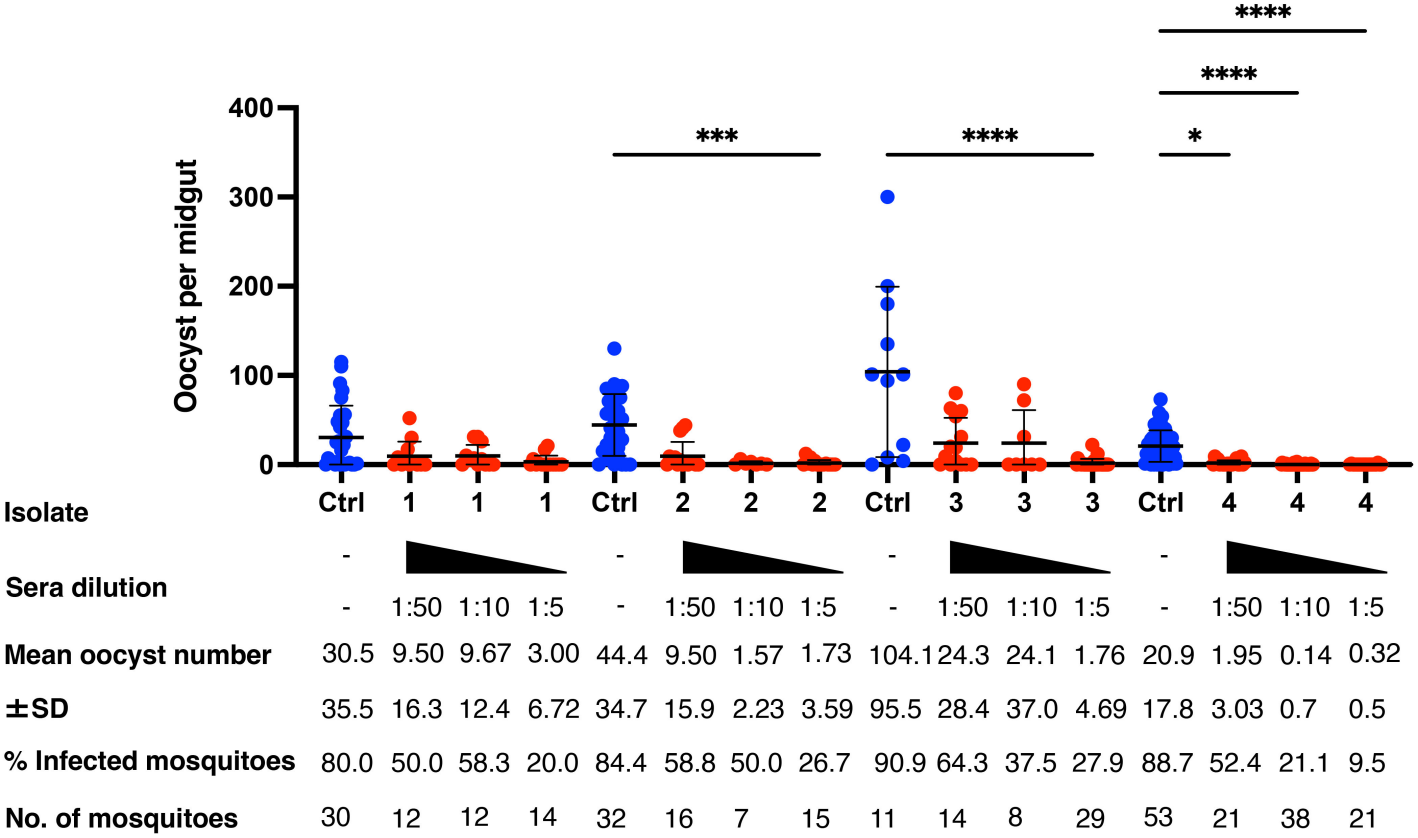
Transmission-blocking activity for *P. vivax* using DMFA. BALB/c mice (n = 5) were immunized with the m8Δ/AAV1-Pv(s25-CSP-VK210/247) vaccine and sera were collected 28 days after the final immunization. Pooled sera were tested using a direct membrane feeding assay (DMFA) involving four *P. vivax* isolates from Brazilian patients (Isolate Nos. 1–4). Oocyst intensity per midgut is shown. Each data point represents the oocyst number from a single blood-fed mosquito (blue dot, control; red dot, immune sera of different dilutions). Horizontal lines indicate the mean number. The mean ± standard deviation, % of infected mosquitoes and number of mosquitoes are summarized below each *P. vivax* isolate. *P*-values were calculated using the Kruskal-Wallis test followed by Dunn’s multiple comparisons test for oocyst intensity of the immunized sample versus the control. *p < 0.05, ***p < 0.001, ****p < 0.0001.

Mosquitoes that fed on the control sera showed a mean intensity of 20.9–104.1 oocysts/midgut, whereas those fed on the immune sera had a mean intensity of 0.14–3.0 oocysts/midgut in a 1:5 dilution. Thus, vaccination achieved a significant transmission reduction activity (TRA) of 96.0% when diluted 1:5, 85.03% when diluted 1:10, and 78.7% when diluted 1:50 for the serum versus blood dilution ratio, although no statistical difference between the immune sera groups was found for the TRA. Furthermore, vaccination achieved a significant TB activity (TBA) of 75.6% (1:5 vs 1:50 *p < 0.05) when diluted 1:5, 59.7% when diluted 1:10, and 34.5% when diluted 1:50 for the serum versus blood dilution ratio (**Figure 4** and **Table 1**). Thus, the vaccine provided potent TB activity with high anti-Pvs25 IgG titers (**Figures 2C, E**).

**Table 1:** TRA and TBA of DMFA.

Immune sera : *P. vivax* patient's blood	Isolate	m8Δ/AAV1-Pv(s25-CSP-VK210/247)		
		1:5	1:10	1:50
TRA	1 2 3 4 **Average**	90.17% 96.09% 98.32% 99.32% **95.98%**	68.34% 96.46% 76.82% 98,49% **85.03%**	68.89% 78.59% 76.67% 90.65% **78.70%**
TBA	1 2 3 4 **Average**	75.00% 68.40% 69.70% 89.30% 75.60%^*^	63.20% 40.70% 58.80% 76.30% **59.75%**	37.50% 30.30% 29.30% 40.90% **34.50%**

Transmission-reducing activity (TRA) and transmission-blocking activity (TBA) are shown for each *P. vivax* isolate (1–4) treated with immune sera (1:5, 1:10, and 1:50 dilutions) as well as their averages. *P*-values were calculated using the Kruskal-Wallis test followed by Dunn’s multiple comparisons test for TRA and TBA. *p < 0.05 (1:5 versus 1:50 at TBA).

## Discussion

Currently, more than 70 *P. falciparum* vaccines targeting different antigens in its complex life cycle are under development and at least 7 of these are undergoing clinical testing (35). Compared with *P. falciparum*, there has been no coherent vaccine program for *P. vivax* (36, 37). This process has been hampered by the lack of a continuous culture system for blood-stages, restricted availability of ideal animal models to study parasite biology, and the lack of funding support (8). Our recent progress in the development of the m8Δ/AAV1-based *P. falciparum* multistage vaccine has enabled us to use it for *P. vivax* vaccines (17). In the present study, the newly developed m8Δ/AAV1-based *P. vivax* vaccine successfully provided 100% protection in a “humanized” rodent malaria parasite model and 95% field-relevant TB efficacy as demonstrated by a DMFA of *P. vivax* isolates from infected patients from the Brazilian Amazon. These findings provide an important proof-of-concept that the m8Δ/AAV1 vaccine platform is sufficiently versatile to be applied to *P. vivax* vaccine development.

In contrast to *P. falciparum* PfCSP, PvCSP is genetically diverse with three major types, VK210, VK247, and the Pv-like variant, which differ in the central repetitive region of the molecule (10). Ideally, a vaccine effective for all three genotypes would be desirable. Our vaccine was designed to express a chimeric PvCSP consisting of the entire PvCSP VK210 and three repeat units of VK247, with the potential to provide worldwide coverage and protection against the predominant *P. vivax* strains. Although the chimeric PvCSP expressed on the surface of cells transduced by the vaccine *in vitro* was recognized by mAbs to the repetitive sequences of VK210 and VK247, mice immunized with the vaccine induced antibodies to the VK210 repeat sequence, but not the VK247 repeat sequence. As a result, the vaccine failed to protect mice against PvCSP VK247 transgenic *P. berghei* sporozoites, which possess the N- and C-terminal conserved regions of VK210 and VK247 and the repeat units of VK247. These results suggest that antibodies to the repeat sequences play a critical role in protection in the murine model. This notion is supported by evidence that the BALB/c (*H-2K^d^*) mice used in our study did not recognize the cytotoxic CD8^+^ cytotoxic T lymphocyte epitopes on the PvCSP sequence. Consistent with previous studies, the protective efficacy of malaria CSP-based vaccines is mainly dependent on antibodies to the repeat sequences, including their titers and avidities, in murine models. In addition to the induction of antibodies to VK210 and VK247, ideal *P. vivax* PEVs would kill all parasites injected by mosquitoes, including sporozoites, those at the liver stage, and hypnozoite parasites to prevent relapses. This requirement appears to be more difficult to achieve for *P. vivax* vaccines than for *P. falciparum* PEVs. T cells targeting infected hepatocytes have the potential to eradicate these cells and thus prevent relapse, indicating our viral-vectored PEV platform capable of inducing cellular immune responses in the *P. falciparum* study is a promising approach for the elimination of *P. vivax* hypnozoites in the liver, although this study did not show any evidence of T-cell-mediated elimination of liver-stage parasites. In the future, we hope to improve the m8Δ/AAV1 vaccine to be effective against the VK210 and VK247 strains by incorporating the full-length CSP sequence of VK247.

Compared with single PEVs, our multistage vaccine will be able to block transmission to mosquitoes even though the vaccine-induced immune responses allow a few pursued remnants to develop hypnozoites in the liver. This could be important in preventing the spread of *P. vivax* infection via the movement of individuals infected with hypnozoites from endemic areas to non-endemic countries where populations lack naturally acquired immunity to *P. vivax* malaria. Our multistage vaccine strategy for *P. vivax* appears to align well with the updated Malaria Vaccine Technology Roadmap to 2030, which advocates for a next-generation vaccine capable of attaining 75% efficacy against *P. falciparum* and *P. vivax* within a two-year timeframe (38). Additionally, the roadmap emphasizes the importance of developing a transmission-blocking vaccine (TBV) targeting sexual stage parasites within mosquito vectors (38). We performed a field study in ILMD-Fiocruz Amazônia, Manaus, Brazil, to evaluate the TB efficacy, as determined by a DMFA using parasites from *P. vivax* isolates from infected patients from the Amazon, an area endemic for *P. vivax* malaria. The DMFA conducted in Brazil utilized blood from four different individuals with varying parasitemia levels among *P. vivax*-infected patients. Furthermore, two species of *An.* mosquitoes, malaria vectors in the Brazilian Amazon basin, were used. Despite differences in parasitemia and the types of *An.* mosquitoes used, consistently high TB effects were demonstrated in all experiments, and reproducibility was achieved. In previous studies, the genetic diversity of Pvs25 observed worldwide was limited, and anti-Pvs25 antibodies were reported to be effective against various *P. vivax* strains without significant impact despite their genetic variations (39). Our m8Δ/AAV1-Pv(s25-CSP-VK210/247) vaccine had 100% protective efficacy against the VK210 strain, and it offers a significant advantage by demonstrating TB effects against the VK210, VK247, and Pv-like strains. However, considering the limited number of mosquitoes used in the DMFA and that patient infection strains have not been identified by PCR, further research will be essential.

In summary, our *P. vivax* multistage vaccine based on the m8Δ/AAV1 platform provided 100% protection and 95.9% TB efficacy (TRA; 1:5 dilution). Moreover, there was no significant difference between dilutions, indicating an effective decrease in transmission even at the highest dilution. Remarkably, the high antibody titers against PvCSP-VK210 and Pvs25 persisted over 8 months and additionally conferred sterile protection against a second challenge in a murine model. Regarding the feasibility of the vaccine for clinical settings, smallpox vaccines are the most successful vaccines in human history and were used worldwide, including Africa, without cold-chain restrictions until 1977. The third-generation smallpox vaccine known as m8, approved as part of Japan’s smallpox immunization campaign, was integral to the World Health Organization’s global smallpox eradication efforts (40). The administration of m8 to over 100,000 children in Japan resulted in an absence of severe vaccine-related adverse effects (41). m8Δ represents a genetically stabilized version of m8, which as a vaccine vector, triggers antibody and cell-mediated immune responses to external antigens with an efficacy approximately 500-fold greater than that of the non-replicating modified vaccinia virus Ankara (40). AAV1 has been already used for gene therapy in humans. Clinical trials are expected to result in equivalent or superior results to those in animal models because m8Δ and AAV1 are human-derived vectors for vaccine and gene therapy, respectively. The safety, efficacy, and production infrastructure of both vaccines are therefore assured. In a subsequent study, we intend to evaluate the safety, immunogenicity, efficacy, and synergistic effects on protection and transmission blockade of this candidate vaccine in a non-human primate model with a view toward Phase I trials.

## Data availability statement

The original contributions presented in the study are included in the article/**Supplementary Material**. Further inquiries can be directed to the corresponding author.

## Ethics statement

All patients with *P. vivax* infection included in the project provided written informed consent to protocols approved by the FMT-HVD ethical board committee (CAAE: 47000221.3.0000.0005, approval number 4.768.634). The studies were conducted in accordance with the local legislation and institutional requirements. The participants provided their written informed consent to participate in this study. All animal care and handling procedures were performed under the approved guidelines of the Animal Care and Ethical Review Committee of Kanazawa University (No. AP-214212), Japan. The study was conducted in accordance with the local legislation and institutional requirements.

## Author contributions

YY: Conceptualization, Data curation, Formal analysis, Funding acquisition, Investigation, Project administration, Writing – original draft, Writing – review & editing. CF: Data curation, Formal analysis, Resources, Writing – review & editing. DO: Data curation, Writing – review & editing. RT: Data curation, Formal analysis, Writing – review & editing. YK: Data curation, Formal analysis, Writing – review & editing. TK: Data curation, Formal analysis, Writing – review & editing. MI: Data curation, Formal analysis, Funding acquisition, Writing – review & editing. AH: Data curation, Writing – review & editing. AS: Data curation, Writing – review & editing. HM: Resources, Writing – review & editing. HS: Resources, Writing – review & editing. SL: Data curation, Resources, Writing – review & editing. SY: Data curation, Formal analysis, Funding acquisition, Resources, Writing – review & editing.

## Supplementary material

Supplementary Figure 1 Experimental design of the sporozoite challenge experiments and TB assays. BALB/c mice (n = 25) were immunized by the indicated regimen and divided into three groups: **(A)** challenged with 1000 PvCSP-VK210/Pb sporozoites at 28 days (n = 10) or 242 days (n = 5) after the final immunization. Protected mice (63 days; n = 10, 277 days: n = 3) were then rechallenged with 1000 PvCSP-VK210/Pb sporozoites 5 weeks after the first challenge, **(B)** challenged with 1000 PvCSP-VK247/Pb sporozoites at 28 days (n = 10) after the final immunization. **(C)** BALB/c mice (n = 10) were immunized by the indicated regimen. Sera from the immunized mice were pooled and a TB assay was performed, as determined by a direct membrane feeding assay using parasites from *P. vivax*-positive, naturally infected donors from the Brazilian Amazon. The time point for boosting immunization with AAV was set as Day 0. Serum collection for the ELISA was conducted one day before each procedure, i.e., immunization, sporozoite challenge, and infection of red blood cells for TB assays, or periodically for long-term monitoring. t.s., tail scaring immunization; i.m., intramuscular immunization; i.v., intravenous injection.

Supplementary Table 1 *P. vivax* profile of patients from Brazil. Parasitemia and gametocytemia are defined as the percentage of parasite-infected red blood cells and gametocyte-infected red blood cells within at least 10,000 observed red blood cells from Giemsa-stained peripheral blood smears, respectively.

Supplementary Table 2 *P. vivax* DMFA methodology. DMFA test samples were divided into four groups: a control group and three test groups (1:5, 1:10, and 1:50 dilutions). All four groups were adjusted to a total volume of 500 µl and used in the experiment.
