# Supplementary material for: A two-dose viral-vectored Plasmodium vivax multistage vaccine confers durable protection and transmission-blockade in a pre-clinical study
Source: Front Immunol. 2024 Apr 30;15:1372584. doi: 10.3389/fimmu.2024.1372584 (PMC11091281; doi:10.3389/fimmu.2024.1372584)
Supplement: Supplementary Figure 1 — Experimental design of the sporozoite challenge experiments and TB assays. BALB/c mice (n = 25) were immunized by the indicated regimen and divided into three groups: (A) challenged with 1000 PvCSP-VK210/Pb sporozoites at 28 days (n = 10) or 242 days (n = 5) after the final immunization. Protected mice (63 days; n = 10, 277 days: n = 3) were then rechallenged with 1000 PvCSP-VK210/Pb sporozoites 5 weeks after the first challenge, (B) challenged with 1000 PvCSP-VK247/Pb sporozoites at 28 days (n = 10) after the final immunization. (C) BALB/c mice (n = 10) were immunized by the indicated regimen. Sera from the immunized mice were pooled and a TB assay was performed, as determined by a direct membrane feeding assay using parasites from P. vivax-positive, naturally infected donors from the Brazilian Amazon. The time point for boosting immunization with AAV was set as Day 0. Serum collection for the ELISA was conducted one day before each procedure, i.e., immunization, sporozoite challenge, and infection of red blood cells for TB assays, or periodically for long-term monitoring. t.s., tail scaring immunization; i.m., intramuscular immunization; i.v., intravenous injection. [file Image_1.pdf]

Supplementary Material

Supplementary Figure S1

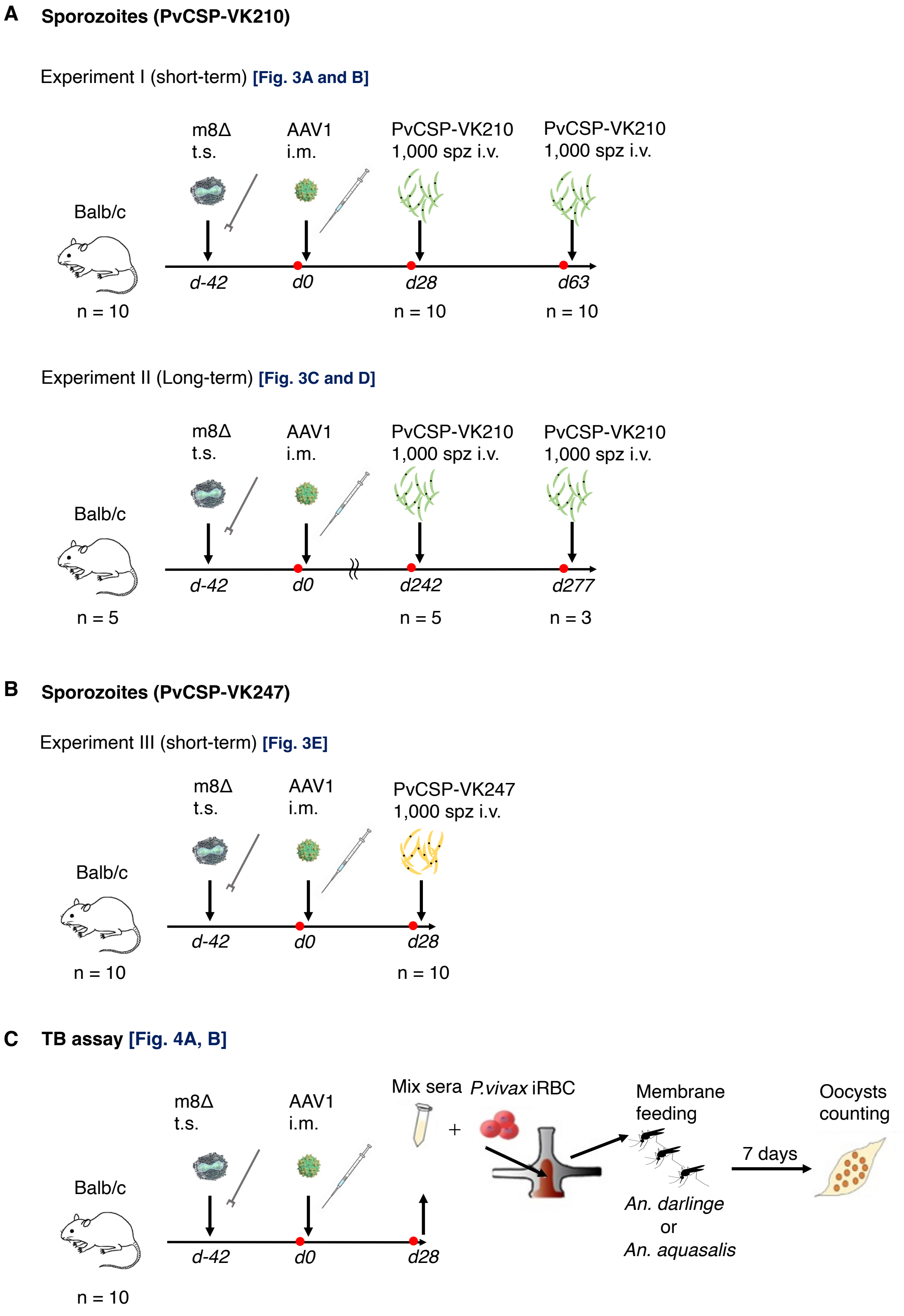

Figure S1. Experimental design of the sporozoite challenge experiments and TB assays

BALB/c mice (n = 25) were immunized by the indicated regimen and divided into three groups: (A) challenged with 1000 PvCSP-VK210/Pb sporozoites at 28 days (n = 10) or 242 days (n = 5) after the final immunization. Protected mice (63 days; n = 10, 277 days: n = 3) were then rechallenged with 1000 PvCSP-VK210/Pb sporozoites 5 weeks after the first challenge, (B) challenged with 1000 PvCSP-VK247/Pb sporozoites at 28 days (n = 10) after the final immunization. (C) BALB/c mice (n = 10) were immunized by the indicated regimen. Sera from the immunized mice were pooled and a TB assay was performed, as determined by a direct membrane feeding assay using parasites from *P. vivax*-positive, naturally infected donors from the Brazilian Amazon. The time point for boosting immunization with AAV was set as Day 0. Serum collection for the ELISA was conducted one day before each procedure, i.e., immunization, sporozoite challenge, and infection of red blood cells for TB assays, or periodically for long-term monitoring. t.s., tail scaring immunization; i.m., intramuscular immunization; i.v., intravenous injection.
