# Supplementary material for: A two-dose viral-vectored Plasmodium vivax multistage vaccine confers durable protection and transmission-blockade in a pre-clinical study
Source: Front Immunol. 2024 Apr 30;15:1372584. doi: 10.3389/fimmu.2024.1372584 (PMC11091281; doi:10.3389/fimmu.2024.1372584)
Supplement: Supplementary Table 1 — P. vivax profile of patients from Brazil. Parasitemia and gametocytemia are defined as the percentage of parasite-infected red blood cells and gametocyte-infected red blood cells within at least 10,000 observed red blood cells from Giemsa-stained peripheral blood smears, respectively. [file Table_1.pdf]

Supplementary Material

Supplementary Table S1

ID	Parasitemia (%)	Gametocytemia (%)
1	0.25	0.045
2	0.07	0.015
3	0.38	0.13
4	0.09	0.0072

Table S1. *P. vivax* profile of patients from Brazil

Parasitemia and gametocytemia are defined as the percentage of parasite-infected red blood cells and gametocyte-infected red blood cells within at least 10,000 observed red blood cells from Giemsa-stained peripheral blood smears, respectively.
