# Supplementary material for: A two-dose viral-vectored Plasmodium vivax multistage vaccine confers durable protection and transmission-blockade in a pre-clinical study
Source: Front Immunol. 2024 Apr 30;15:1372584. doi: 10.3389/fimmu.2024.1372584 (PMC11091281; doi:10.3389/fimmu.2024.1372584)
Supplement: Supplementary Table 2 — P. vivax DMFA methodology. DMFA test samples were divided into four groups: a control group and three test groups (1:5, 1:10, and 1:50 dilutions). All four groups were adjusted to a total volume of 500 µl and used in the experiment. [file Table_2.pdf]

Supplementary Material

Supplementary Table S2

	Control	1:5	1:10	1:50
The patient’s infected Red blood cells	250 µl	250 µl	250 µl	250 µl
Inactivated Antibody human serum	150 µl	150 µl	200 µl	240 µl
Non-immunized mouse serum (Control)	100 µl	-	-	-
Immunized mouse serum (Test)	-	100 µl	50 µl	10 µl
Total	500 µl			
Hematocrit	50%			

Table S2. ***P. vivax*** **DMFA methodology**

DMFA test samples were divided into four groups: a control group and three test groups (1:5, 1:10, and 1:50 dilutions). All four groups were adjusted to a total volume of 500 µl and used in the experiment.
